# MissMax: alignment-free sequence comparison with mismatches through filtering and heuristics

**DOI:** 10.1186/s13015-016-0072-x

**Published:** 2016-04-21

**Authors:** Cinzia Pizzi

**Affiliations:** Department of Information Engineering, University of Padova, via Gradenigo 6/a, 35131 Padova, Italy

**Keywords:** Sequence similarity, Alignment free, Mismatches, Compositional approaches, Phylogenetic analysis

## Abstract

**Background:**

Measuring sequence similarity is central for many problems in bioinformatics. In several contexts alignment-free techniques based on exact occurrences of substrings are faster, but also less accurate, than alignment-based approaches. Recently, several studies attempted to bridge the accuracy gap with the introduction of approximate matches in the definition of composition-based similarity measures.

**Results:**

In this work we present *MissMax*, an exact algorithm for the computation of the longest common substring with mismatches between each suffix of a sequence *x* and a sequence *y*. This collection of statistics is useful for the computation of two similarity measures: the longest and the average common substring with *k* mismatches. As a further contribution we provide a “relaxed” version of MissMax that does not guarantee the exact solution, but it is faster in practice and still very precise.

## Background

Sequence similarity has long been playing a crucial role in Computational Biology and Bioinformatics as a key ingredient in the prediction of functional and structural properties, and of evolutionary mechanisms.

Since the introduction of high throughput techniques, hundreds of fully sequenced genomes of different species have been made available at a fast pace. The increasing number of available sequences makes all kind of sequence analysis, most notably assembly, phylogenetic reconstruction, and multiple alignments, more challenging due to the time consuming and memory-demanding operations that need to be carried out on these huge datasets.

To try to cope with the increasing demand of time efficiency, a wide range of alignment-free (or composition-based) approaches have been proposed. The idea behind compositional approaches is to model each sequence in terms of the substrings that it contains, and then to devise appropriate similarity measures to compare two sequences based on this model [19].

Traditionally, alignment-free approaches rely on the frequency or presence of *L*-mers, for a fixed length *L*, and consider exact matches. Although usually very fast, in several contexts such approaches can be much less accurate than alignment-based counter-parts.

For this reason, within the last decade, several approaches have been proposed to improve the ability to better capture the nature of the similarity/dissimilarity between biological sequences with alignment-free techniques. Among the wide literature, we can mention, for example, the introduction of over-representation, rather than the raw frequency count, in the definition of the similarity measure for fixed length [17] and maximal length [3, 4] components; and the definition of distances based on average longest shared substrings [18], which frees the analysis from fixing the length of the substrings to analyse.

More recently, several studies proposed to model the intrinsic variability of biosequences by considering approximate matches with a bounded number of mismatches, or by using spaced-words, in the characterization of the sequence composition. Several related experiments showed that, in the context of phylogenetic tree reconstruction, the introduction of approximate matches can improve the quality of the detected sequence similarity [5, 13, 14].

Given these premises, we focused our attention on these more involved formulations of the alignment-free approach, in particular on those allowing for approximate matching within a bounded number of mismatches.

As a warm up we will give a brief overview of recently proposed approaches, and will use some of the presented results to introduce further notation used throughout the paper.

Let us consider two sequences $x=x_1\dots x_n$ and $y=y_1\dots y_m$ defined over an alphabet $\Sigma$. Let $X_i=x_i \dots x_n$ and $Y_j=y_j\dots y_m$ be the suffixes of *x* and *y* starting at position *i* and *j* respectively. In the following we will assume, without loss of generality, that both sequences have the same length *n*.

An early result on speed-ups for the computation of empirical statistic with mismatches was presented in [15], where an $O(n^2)$ algorithm was proposed to compute the number of occurrences with *k* mismatches of all the substrings of length *L* in a string *x* of length *n*. The key feature of this algorithm is that its complexity is independent on the number of mismatches that are allowed. The algorithm was proposed within the pattern discovery framework [7, 8], thus the need to count the occurrences within the same string in order to subsequently estimate their over-representation. However, the proposed solution can be easily adapted to compute the number of occurrences of all the substrings of length *L* of a string *x* in another string *y*, leading to the definition of a similarity measure between the two sequences based on the frequency of shared approximate occurrences.

The first formal definition of similarity measures based on shared *maximal* substrings with mismatches was introduced in [5]. We report here the main concepts, but with a slightly different notation. Let $LCP_k(x,y)$ be the length of the longest common prefix between two strings *x* and *y* when *k* mismatches are allowed. Now, consider the set of $LCP_k(X_i,Y_j)$ defined for all the suffixes $X_i, i = 1,2,\ldots , n$ of *x*, and for all the suffixes $Y_j, j =1,2,\ldots , n$ of *y*.

The following measures of cross correlation were defined for a given number of mismatches *k*:

### **Definition 1**$MaxCor_k(i)$

$MaxCor_k(i), i = 1, 2, \ldots , n$, is an *n*-length vector storing the maximum value attained by $LCP_k(X_i,Y_j)$ for each *i* over all values correspondingly spanned by *j*.

### **Definition 2**$AvgCor_k(i)$

$AvCor_k(i), i = 1, 2, \ldots , n$, is an *n*-length vector storing the average value attained by $LCP_k(X_i,Y_j)$ for each *i* over all values correspondingly spanned by *j*.

### **Definition 3**$MaxCor_k$

$MaxCor_k$ is the maximum value attained by $LCP_k(X_i,Y_j)$ over all values of $i \in (1,2,\ldots , n)$ in *x* and $j \in (1,2,\ldots , n)$ in *y*.

### **Definition 4**$AvCor_k$

$AvCor_k$ is the average value attained by $LCP_k(X_i,Y_j)$ over all values of $i \in (1,2,\ldots , n)$ of *x* and $j \in (1,2,\ldots , n)$ in *y*.

For measures such as $MaxCor_k$ and $AvCor_k$ in [5] it was also proposed a subquadratic algorithm for their computation. This can futher be improved to $O(\frac{n^2}{\log n})$ [6].

In [14] *kmacs*, a greedy heuristic, was proposed to generalize the well known Average Common Substring (ACS) distance [18] so to account for *k* mismatches when considering the longest common substring between pairs of positions in the two strings. We refer to this variant of the ACS problem as *kACS*. The algorithm proposed in [14] has time complexity *O*(*nkz*), where *z* is the maximum number of occurrences in *y* of a string of maximal length occurring in both *x* and *y*. Being based on a heuristic, this method is very fast in practice, but it does not guarantee to find the optimal solution to the problem. Note that the kACS problem can be described in terms of the measures of cross correlation previously defined as the mean over all positions *i* in *x* of $MaxCor_k(i)$.

We end our overview with some recently published theoretical results. In [9] the *k*-LCF problem is introduced as the generalization of the longest common substring (there named “factor” to avoid confusion with LCS as the Longest Common Subsequence problem) as finding the longest common shared match between two sequences when up to *k* mismatches are allowed. Also this problem can be described in terms of the previously defined scheme, as it corresponds to $MaxCor_k$. In [9] an *O*(*nm*) time and *O*(1) space solution is provided for a generic *k* and two strings of length *n* and *m* respectively, and also an $O(n+m)\log (n+m)$ solution for the case $k=1$. Finally, in [2] an $O(n \log ^{k+1} n)$ time and *O*(*n*) space algorithm was proposed to provide a subquadratic solution to the kACS problem.

Within this framework we developed a new strategy based on filtering for the computation of the longest common substring with mismatches between all the suffixes of a sequence *x* and another sequence *y*. This primitive is at the basis of recently proposed similarity measures for the problem of phylogentic tree reconstruction as the longest and the average common substring distances with mismatches.

Formally, we will compute the values of $MaxCor_k(i)$, for all $i=1\dots n$, because this vector allows us to derive the values of both $MaxCor_k$ (or equally *k*-LCF) and of *kACS*, being respectively:

1 $$\begin{aligned} MaxCor_k = \max _{i=1\dots n} {MaxCor_k(i)} \end{aligned}$$

2 $$\begin{aligned} kACS = \frac{1}{n} \sum _{i=1\dots n} {MaxCor_k(i)} \end{aligned}$$

The paper is organized as follows: in "Methods" section we will describe the proposed filtering-based approach to compute the values of all $MaxCor_k(i)$. In "Results and discussion" section we will discuss the results of a set of experiments we devised to test the performances of the proposed algorithms in practice. In "Concluding remarks" section we will lead to the conclusions.

## Methods

Our aim is to compute the values of $MaxCor_k(i)$ for each position *i* in *x*. The main idea behind the proposed approach is to avoid the computation of the $LCP_k(X_i,Y_j)$ for all pairs of positions $i \in x$ and $j \in y$. To this purpose we will compute the value of $MaxCor_k(i+1)$ starting from the value of the already computed $MaxCor_k(i)$. This procedure will initially give us a candidate longest match $L_{max}$ that is at least equal to $MaxCor_k(i-1)-1$. We will use this information, among some others that will be discussed in the following subsections, to reduce the cardinality of the set $\mathcal {C}$ of possible candidates for approximate matches longer than $L_{max}$, and then we will verify them.

Note that, when computing the $MaxCor_k(i)$ for each *i*, one can either take track of their maximum value to compute $MaxCor_k$, or of their sum to later compute kACS at no extra cost.

In the description of the algorithm we will refer to techniques that allow us to keep the worst case analysis within the claimed worst case quadratic bound, but for practical purposes we will use different approaches that will be discussed in a dedicated subsection.

### Initial set up: $MaxCor_k(1)$

We start with the computation of $MaxCor_k(1)$ as the maximum approximate match with *k* mismatches of the suffix $X_1$ against the sequence *y*. For this purpose we will start from, and exploit, the classical concept of longest common substring (without any mismatch allowed).

#### **Definition 5***Longest common substring*

Given two sequences *x* and *y*, of length *n*, find the maximum length *L* for which a pair of indexes (*i*, *j*) exists such that $x_i \dots x_{i+L-1} = y_j\dots j_{j+L-1}$.

The problem of finding the longest common substring between two sequences is a well known problem in pattern matching that can be solved in linear time by the traversal of a generalized suffix tree of the two sequences. More in details, we want to be able to find the longest common match starting at any two positions *i* in *x* and *j* in *y*. This problem can be solved through a call to the Lowest Common Ancestor of the corresponding leaves $n_i$ and $n_j$ in the generalized suffix tree. The length of the label of the path from the root to $LCA(n_i,n_j)$ is the length of their longest common prefix. LCA queries can be carried out for any *i* and *j* in constant time after a linear-time preprocessing step [10].

In particular, similarly to the routine step in [14], we will perform $k+1$*jump-extensions* to compute the longest approximate match between $X_1$ and the generic $Y_j$. As after the first jump-extension of length $l_1$ we know we will have a mismatch, we will call LCA on the nodes corresponding to positions $1+l_1+1$ and $j+l_1+1$, and repeat the procedure until the $(k+1)$-th mismatch is found. This is repeated for each $j=1\dots n$, thus taking *O*(*kn*) time overall.

### Minimum $MaxCor_k$ from the previous step

**Fig. 1:**
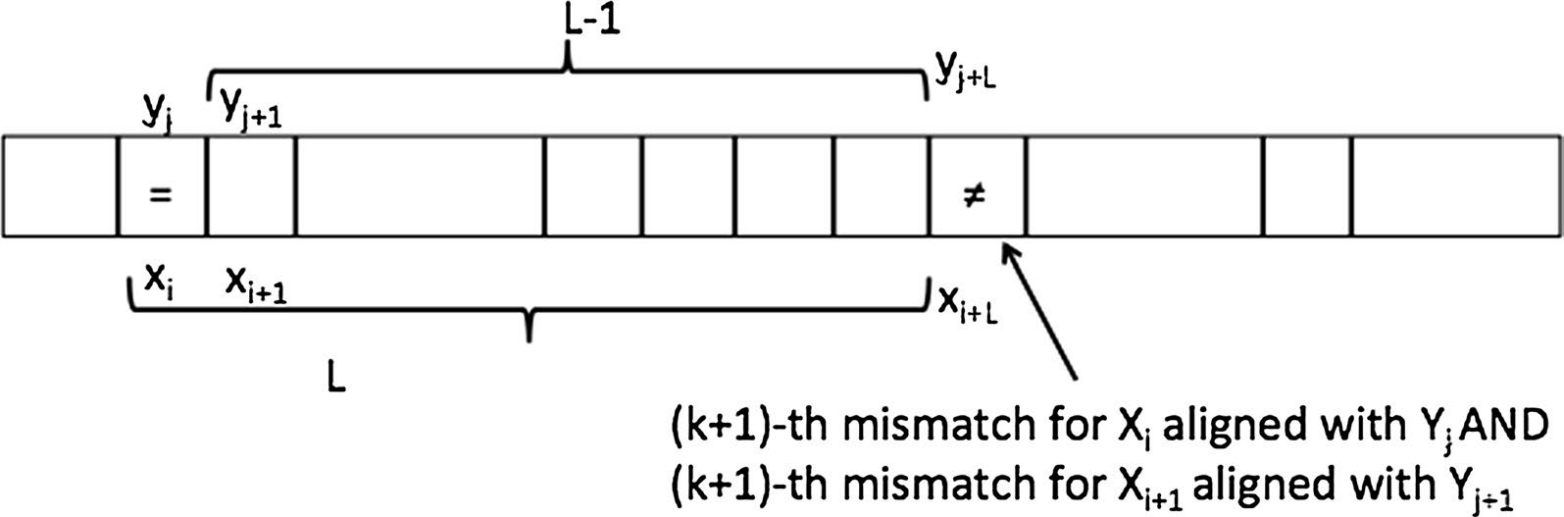
Candidate $MaxCor_k(i+1)$ from $MaxCor_k(i)$ when $x_i = y_j$

**Fig. 2:**
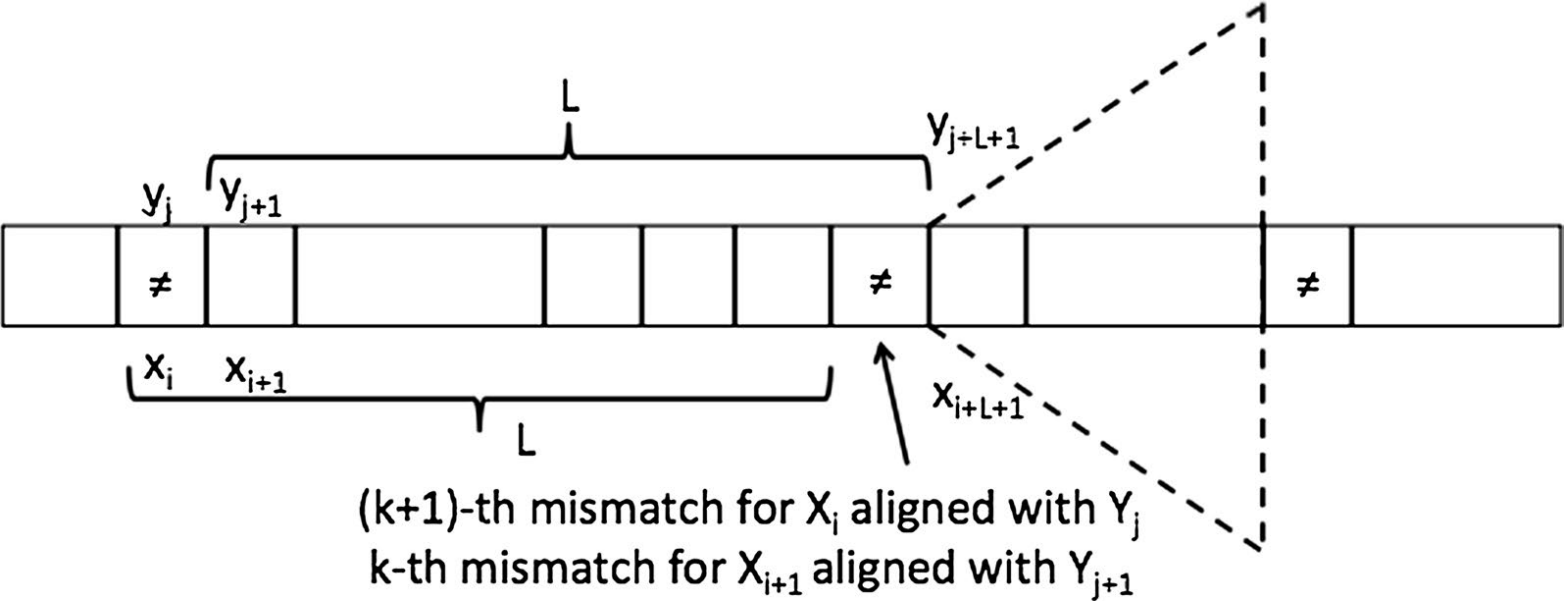
Candidate $MaxCor_k(i+1)$ from $MaxCor_k(i)$ when $x_i \ne y_j$

Assume now we have computed $L=MaxCor_k(i)$, and we want to compute $MaxCor_k(i+1)$. Let *j* be the position in *y* of a longest approximate match of $X_i$. Two cases may hold, which are illustrated in Figs. 1 and 2 respectively:

1. $\mathbf{x_i = y_j}$: in this case the *k* mismatches all lie within $x[i+1,i+L-1]$ and $y[j+1,j+L-1]$, respectively. Therefore we have $LCP_k(X_{i+1},Y_{j+1})=L-1$. Note that this might or might not be the final $MaxCor_k(i+1)$ over all positions of *y*.
2. $\mathbf{x_i \ne y_j}$: in this case the mismatch between the first characters will be lost when considering the alignment of $i+1$ and $j+1$, leading to $k-1$ mismatches in the following $L-1$ positions. After *L* positions we know we must have a mismatch, which is now counted as the *k*-th. To finally obtain $LCP_k(X_{i+1},Y_{j+1})$ we need a further call to LCA on the nodes corresponding to the positions $i+L+1$ and $j+L+1$ to obtain $LCP_0(X_{i+L+1},Y_{j+L+1})$ that will end on the $(k+1)$-th mismatch. In summary: $LCP_k(X_{i+1},Y_{j+1})=L+LCP_0(X_{i+L+1},Y_{j+L+1})$. Again, note that this might or might not be the final $MaxCor_k(i+1)$ over all positions of *y*.

It is possible that several suffixes in *y* are the site of a longest match with *k* mismatches with $X_i$. All the starting positions of these longest matches are considered for further extension at this step. This is done for two purposes:

- to obtain the longest possible candidate length with *k* mismatches from the previous step at the minimum cost (only a jump till the next mismatch to the right is needed to have the exact length for the pairs of positions considered at this step);
- to avoid or reduce the possibility that we have to deal with special cases in the following steps (see Observation 4 in "Theoretical and Practical Considerations" section).

Let $L_{max}$ be the candidate value for $MaxCor_k(i+1)$ obtained either from Case 1 or Case 2.

### Potential candidates from the previous step

Let us consider now a generic position *r* in *Y*. We must have $L' = LCP_k(X_i,Y_r) < L$, since *L* was the absolute maximum found in the step to compute $MaxCor_k(i)$, and the ties have already been considered.

If $\mathbf{x_i = y_r}$ then *k* mismatches lie between $x[i+1,i+L'-1]$ and $y[r+1,r+L'-1]$, respectively, and $LCP_k(X_{i+1},Y_{r+1})=L'-1 < L-1$. As a consequence, the pair $(i+1,r+1)$ can be ruled out as one that cannot have an approximate match longer than the one we are currently considering (which is greater or equal than $L-1$). Note that this observation allows us to exclude from the candidate set $\mathcal C$ all the positions $r+1$ in *y* that are preceded by a symbol matching $x_i$.

**Fig. 3:**
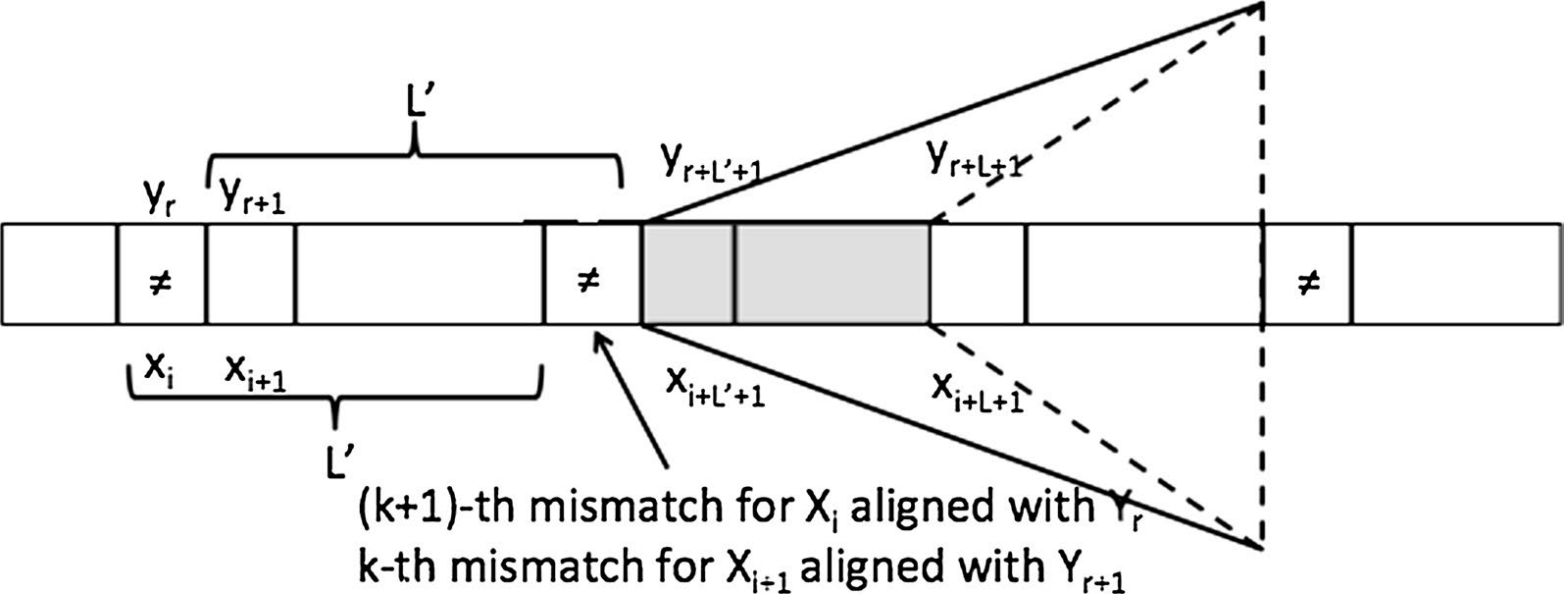
Guessing the maximum extension between the suffix $X_i$ and a candidate $Y_r$

The case where $\mathbf{x_i \ne y_r}$ is more involved. With reference to Fig. 3, the alignment $(X_{i+1},Y_{r+1})$ loses the mismatch in the first position of the alignment $(X_i,Y_r)$, and includes the one at position $i+L'$ and $r+L'$, in *x* and *y* respectively. To obtain the length of $LCP_k$ for this alignment we should add to $L'$ the value of $LCP_0(X_{i+L'+1},Y_{r+L'+1})$, which gives the last exact contribution till the $(k+1)$-th match. It may happen that the addition of this term to $L'$ allows one to obtain a match longer than the potential $MaxCor_k(i+1)=L_{max}$ we had from the previously discussed Case 1 or Case 2. The main problem here is that we do not know the value of $L'$.

We will then proceed by assuming *r* is indeed the site of a match longer than the current maximum $L_{max}$. If this is the case, the gap with $L_{max}$ must be closed assuming the $(k+1)$-th mismatch occurs after the positions $i+L$ and $r+L$ in the two strings. As a consequence, $LCP_0(X_{i+L+1},Y_{r+L+1})$ will end exactly where $LCP_0(X_{i+L'},Y_{r+L'})$ would end (see Fig. 3).

If this value is indeed bigger than or equal to $L_{max}$ we need to make sure no further mismatch was present between $i+L'$ and $i+L$. This can be checked by running the jump-extension performed in the initial setup starting from positions $i+1$ and $r+1$ until $k+1$ mismatches are found. Let $L_{true}$ be the reached extension. If its value is equal to $L+LCP_0(X_{i+L+1},Y_{r+L+1})$ then the position *r* is the new candidate position for the longest match of the suffix $X_i$ in *y*, and the list of ties is reset, otherwise the position is dropped, and the next candidate is considered. If $L+LCP_0(X_{i+L+1},Y_{r+L+1})$ is equal to $L_{max}$, then *r* is added to the list of ties.

### Theoretical and practical considerations

We now discuss some theoretical and practical issues emerging from the proposed approach.

*Observation 1.* The worst case complexity occurs when we inherit from step *i* an initial candidate that is smaller or equal than $MaxCor_k(i)$. In such a case any position *r* for which $x_{i+L_{max}+1}=y_{r+L_{max}+1}$ is a possible longer match that we need to verify with the jump-extension. This lead to potential *O*(*n*) candidate pairs per position *i*, and to a worst case time complexity $O(kn^2)$.

*Observation 2.* We observe that even in the worst case, we can rule out all the positions $j+1$ of *y* that are preceded by a character that matches $x_i$. Assuming equal distribution, we will drop $\frac{n}{|\Sigma |}$ positions. In practice, we will drop a number of position equal to the frequency of $x_i$ in *y*.

*Observation 3.* Whenever we impose a matching condition we potentially reduce the candidate set of a fraction equal to $\frac{|\Sigma |-1}{|\Sigma |}$. When searching for a match longer than the current one we have that at least the first symbol of the last jump must be a match in order to have a longer match. Furthermore, if the value of $L_{max}$ computed from the longest matches at the previous step is longer than $L-1$ (which is the minimum) we can impose a match for the whole segment $[x_{i+L}\dots x_{i+L_{max}-1}]$, thus further reducing the size of the candidate set.

*Observation 4.* At step $i+1$, if the value of $L_{max}$ obtained from the longest matches at step *i* is $L-1$ there is the possibility that the actual longest match for $X_{i+1}$ is smaller than the one at the previous step. Searching for ties when the longest match is $L-1$ is a very time consuming operation because we cannot apply the reduction of the candidate positions explained in Observation 3. This is because when $L_{max}=MaxCor_k(i)-1 = L-1$ and $x_i \ne x_r$ we will have that $x_{r+L}$ lands on the *k*-th mismatch rather than on the first position after that, thus ending up to erroneously discard position $r+1$ from further processing. For this reason we will always try first to find a strictly longer match. If we cannot find it, we will search for ties at length $L-1$ using only Observation 1 (and excluding of course also the positions that were already processed).

*Observation 5.* The case discussed in Observation 4 is very time consuming because the candidate set remains pretty large. However, we have verified in practice that the number of ties that are actually found during that step is very small, and orders of magnitude less than the size of $\mathcal C$. For this reason we develop an “relaxed” version of our algorithm in which we ignore the search of ties for the case $L-1$. This means that we are no longer guaranteed to find the longest match for each suffix $X_i$, but in practice in our experiments the error was always negligible, and the time needed for the computation was reduced of about a half as it will be shown in "Results and discussion" section.

*Observation 6.* Building indexing data structures can be expensive, and so can be operations that are theoretically efficient. For example, it was already observed in [14] that a naive extension to account for *k* mismatches gave better performances than calling LCA (or than performing the equivalent operation on an enhanced suffix array [1], as they did). In our experiments we experienced the same. Moreover, in [12] it is shown that, in practical applications, a simple computation of the longest common extension (LCE) between two strings can substantially improve the performances of several algorithms that use the LCE as a subroutine. Therefore, by keeping the original approach in mind, but avoiding reference to indexing data structures, we developed a tool, implementing our algorithm *MissMax*, in which the extensions are performed naively. Note that in many cases we just need to perform a one-step extension, as the *k*-step extensions are performed only in the initial step, and whenever we have a candidate with an approximate match longer than $L_{max}$.

*Observation 7.* In its current implementation MissMax uses $|\Sigma |$ arrays of bits to take track of the presence or absence of symbols at specific positions in *y*, in order to quickly compute the set of candidates for further inspection. For DNA applications, when reading the input, we will build 4 arrays $A_s$, one for each symbol $s \in \Sigma$. The position *i* of $A_s$ is set if and only if $y_i=s$. When computing $MaxCor_k(i+1)$, the first filter is given by the complement of $A_{x_{i}}$. To get the second filter we take the array $A_{x_{i+L}}$ and shift it of *L* positions to the left. The bitwise AND of the two vectors is the bitvector *B* mentioned above that holds the positions of the candidate set $\mathcal C$ (to avoid a further shift of one position to the right, when considering position *j* we look at the value of this vector at position $j-1$). This particular implementation limits the applicability to genomic sequences. However, this is not a theoretical limit of the approach. By changing the data structures used to store the sequences, and the approach to candidate identification, it will be also possible to deal with larger alphabets. For this purpose we plan in the near future to develop a library to compute statistic with mismatches on all kind of biological sequences.

## Results and discussion

In this section we present the results of a set of experiments that we run to test the performances of the exact and relaxed version of MissMax. Here we are not mainly interested in the improvement of the quality of the tree reconstruction with mismatches, with respect of corresponding measures without mismatches, as they were already discussed in [5, 14]. We are rather interested on the time needed to compute the values of $MaxCor_k(i)$, for all the positions *i* in a sequence *x* with respect to a second sequence *y*, and on the precision achievable by heuristics. As explained in "Methods" section, these statistics can be used to compute both the $MaxCor_k$ (i.e. the longest common substring with *k* mismatches) based distance discussed in [5], and the kACS distance discussed in [14]. Moreover, as our algorithms are based on filtering, we will investigate also the filtering power of our approach for both the exact version and relaxed version discussed in Observation 4.

For what concerns the comparison with other algorithms, the algorithm described in [2], which holds the best known asymptotic complexity for the exact computation of the *n* values of $MaxCor_k(i)$, has no available implementation yet. For the exact computation we will thus refer to the naive algorithm. On the other side of the spectrum, the greedy-based approach of kmacs [14], is uncomparably fast in almost all cases (we will discuss in details one experiment in which this did not occur). With respect to this approach we will therefore focus our attention on the precision achievable in the estimate of the actual value of the longest matches.

For our experiments we considered two datasets that were previously used in other studies (e.g. [5, 14, 18]). The first dataset consists of the mitochondrial genomes of 34 mammals, including species from Euarchontoglires, Laurasiatheria, Afrotheria, Xenarthra, Ameridelphia, and Monotremata. The second dataset consists of the mitochondrial genomes of 27 primates. The length of the genomes is between 16,000 and 17,000 bp each. All the sequences were downloaded from the NCBI web site. All the experiments were performed on an Intel Core i5-4590 at 3,3 GHZ, with 8 GB of memory.

### Time performances

As a first experiment we measured the time performances of both the exact and the “relaxed” version of MissMax on omogeneous and heterogeneous subsets of the 34 mammals datasets. In particular, we considered the subset of Rodents (rat, dormouse, house mouse, guinea pig, squirrel), the subset of Carnivora (cat, dog, harbor sail, grey sail), and a mixed set of the two. We then measured the average time needed to compute the similarity between two sequences within each of the omogeneous set, and between elements of different sets in the mixed subset. Finally we added to our analysis a set of 5 random sequences and measured the performances within the subset. Tables 1 and 2 report, for different *k*, the average time for the comparison of a pair of sequences in each dataset for the relaxed and the exact version of MissMax, respectively.

**Table 1:** Average time (in seconds) for the comparison of two sequences on several datasets with the “relaxed” version of MissMax

k	Rodents	Carnivora	Mixed	Random
5	2.11	2.22	2.15	1.85
10	2.45	2.56	2.47	2.18
20	3.07	3.17	3.09	2.72
50	4.74	4.87	4.82	4.28
100	7.42	7.62	7.57	6.65

**Table 2:** Average time (in seconds) for the comparison of two sequences on several datasets with the exact version of Missmax

k	Rodents	Carnivora	Mixed	Random
5	4.50	5.16	4.45	3.26
10	5.16	6.39	5.64	3.81
20	7.47	8.46	7.52	4.85
50	12.08	14.29	12.9	8.26
100	21.01	23.55	21.28	13.15

It is immediate to observe that the relaxed version of MissMax takes about half of the time needed by the exact version. Nevertheless, even the exact algorithm allows for a full pairwise comparison of typical datasets in about an hour on our desktop computer. The time required for each comparison is consistent with the one showed for the smaller datasets, thus the time required for the overall analysis is a function of the size of the dataset. For example, with $k=5$ the analysis of the full dataset of 27 primates took about 55 min, while the full analysis of the 34 mammals dataset took 1 h and 24 min. Moreover, if we take advantage of multithreading, the full analysis of the Primate dataset requires about 15 min, and the full analysis of the Mammals dataset about 23 min.

With respect to the kind of sequences that are analized, we can see how both algorithms are faster on the random dataset than on the biological datasets. The difference is much more evident for the exact version of the algorithm, when *k* increases.

For comparison with other approaches, we run the naive algorithm on the Rodents datasets. The average time was: 7.1 s for $k=5$; 10.6 s for $k=10$; 17.8 s for $k=20$; 39.3 s for $k=50$; and 74.99 s for $k=100$. On the contrary, the software *kmacs* is generally much faster than MissMax: on such small datasets it ends practically istantaneously. We recall that the theoretical time complexity of kmac is *O*(*kzn*), where *z* is the number of positions in which there is a tie for the first longest exact match. It is shown in [14] that in practice the values of *z* are usually pretty small. Nevertheless, we report, for completeness of discussion, the following observation. While running our experiments, we had a case in which, increasing the length of the sequences to compare, kmacs performances had a suddent drop. After ruling out this could depend on the actual input length of the sequences (the performances were good on random sequences of the same length), or on some bug (the results were what we were expecting), a closer inspection of the input sequences revealed the probable cause. More or less in correspondance of the input length that was showing the slow down, there was an undefined region in one of the two sequences. Note that the computed value of the longest matches of suffixes was not affected, as the *N* region was present just in one sequence. Nevertheless, the first exact longest match of a suffix starting in the *N* region is 0, and in such a case, *z* becomes equal to *n*, and all the positions of the other sequence need to be extended to find the maximum extension with mismatches.

**Fig. 4:**
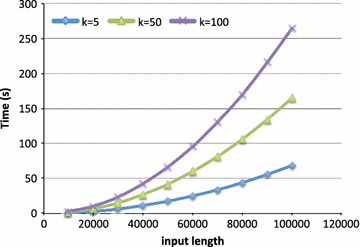
Time performance of relaxed MissMax for different values of *k*, as a function of the input length

Finally, to further investigate the scalability of the filter-based approach we performed a similar test on longer sequences. The results are reported in Fig. 4, where we show the time required for three different values of *k*, on sequences up to 100k bp, as a function of the input length. The trends for the exact version are the same. We reported a slowdown of about 2, with the actual values slightly increasing with *k*. Specifically, the average slowdown factor was 1.87 for $k=5$; 2.22 for $k=50$; and 2.29 for $k=100$.

### Exact vs heuristics

The tradeoff between exact algorithms and heuristics could be easily summarized saying that heuristics are faster, but do not guarantee the correct solution to the problem. This is of course true also in our case, but we performed anyway some further analysis to assess the performances of MissMax (both exact and relaxed version) and kmacs, in terms of precision in the computation of sequence similarity based on the aforementioned distances with mismatches.

**Fig. 5:**
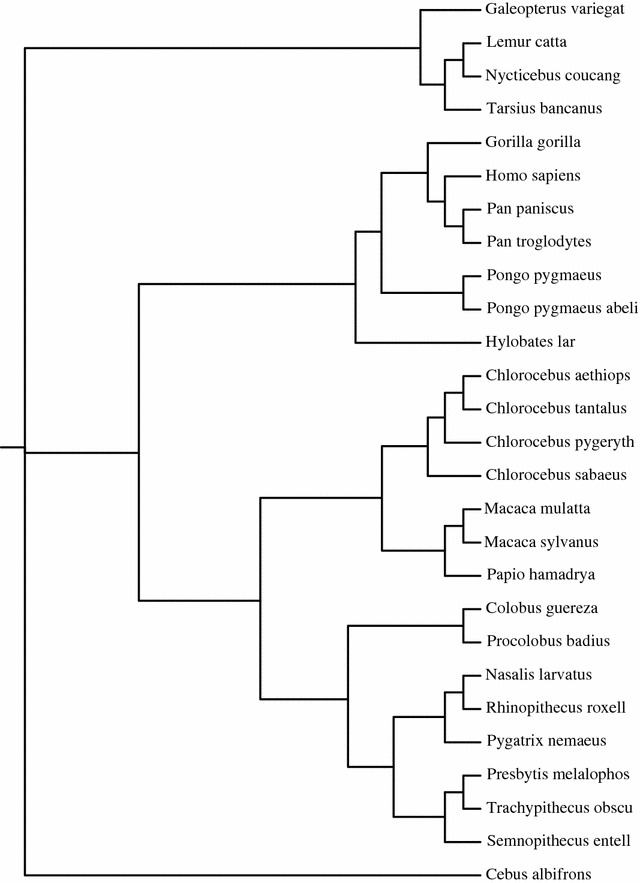
The tree for the 27 primates dataset reconstructed by MissMax with $k=4$. It is in perfect agreement with the reference tree reported in [11]

In terms of reconstruction of the correct phylogeny, we tested the algorithms on the Primates datasets. MissMax reported the same reference tree as in [11] (see Fig. 5; the permutations within a same level of the tree are considered as equivalents) for a relatively small number of allowed mismatches ($k=4$ and $k=5$). kmacs reported an overall good reconstruction, but with some differences with respect to the references (in [14] a wide range of *k*’s have been tested on the same dataset, but the exact reference phylogeny was never captured).

As previosuly stated, kmacs is usually much faster even than the relaxed version of MissMax. However, in terms of computation of the actual value of the average common substring between two sequences, relaxed MissMax is much more precise. In fact, we measured a relative error of 0.53 % with respect to the real measure for both the Rodents and the Carnivora datasets, and 0.46 % for the mixed dataset. For the full Primate dateset, we measured the relative error for both $k=5$ and $k=10$, reporting 0.45 % and 0.43 % respectively. kmacs did not achieve such a precision in the approximation of the correct value. In our experiments it usually estimates half of the length the average common substring. This lack of precision was noted also by the authors of kmacs in a set of experiments reported on their paper (although on simulated sequences with a given error rate). Anyway, as reported in [14], this does not seem to heavily affect the reconstruction of a phylogenetic tree. This may be due to the fact that the understimation holds equally for all pair of sequences. However, if the statistics need to be collected for other kind of analysis, then one has to keep in mind that the approximation provided by kmacs could not be as good as the one provided by relaxed MissMax.

### Filtering power

**Table 3:** Average percentage of pair of positions considered in several datasets with the relaxed filter, with respect to the quadratic maximum number of pairs

Relaxed MissMax	k = 5	k = 10	k = 20	k = 50	k = 100
Rodents	7.26	8.37	9.15	9.74	9.93
Mixed	7.16	8.15	8.76	9.28	9.79
Random	9.73	13.31	15.17	15.29	15.27

**Table 4:** Average percentage of pair of positions considered in several datasets with the exact filter, with respect to the quadratic maximum number of pairs.

Exact MissMax	k = 5	k = 10	k = 20	k = 50	k = 100
Rodents	9.16	12.38	16.08	18.13	19.89
Mixed	9.34	12.64	16.45	17.93	19.32
Random	15.40	26.92	33.51	32.56	31.54

A set of experiments was performed to measure the goodness of our filters, investigating on the percentage of pair of positions that are actually considered with respect to the maximum possible. The results are shown in Table 3 for the relaxed and in Table 4 for the exact version of MissMax.

We note that in the biological sequences the difference between the size of the candidate set of the relaxed and exact version is quite small for $k=5$, but then increases substantially with *k* up to $k=50$, and then remains pretty much constant for $k=100$. The random sequences follow the same trend, but they appear to reach the saturation level earlier at $k=20$.

It is interesting to note that the analysis on random sequences is faster than in biological sequences (see Tables 1, 2) although the size of the candidate set is bigger. We explain this behavior as due to the time needed to make full check for a match. The comparison is stopped either when the expected length is reached or when the number of mismatches is $k+1$. It is possible that with random sequences the second condition occurs more often after few comparisons, thus speeding up the entire process, even if the number of positions to check is bigger.

## Concluding remarks

In this work we proposed a filtering-based approach for the computation of the longest common substring with *k* mismatches between *each* suffix of a sequence *x* and a sequence *y* we want to compare to. This statistics is useful for the computation of alignment free distances based on approximate matching, that are a promising approach to improve the quality of alignment free sequence comparison. We developed both an exact and a relaxed version of the algorithm. While the relaxed version cannot guarantee to find the optimal solution, it is in practice faster and still very precise.
